# Exploitation of Eukaryotic Ubiquitin Signaling Pathways by Effectors Translocated by Bacterial Type III and Type IV Secretion Systems

**DOI:** 10.1371/journal.ppat.0030003

**Published:** 2007-01-26

**Authors:** Aurélie Angot, Annette Vergunst, Stéphane Genin, Nemo Peeters

## Abstract

The specific and covalent addition of ubiquitin to proteins, known as ubiquitination, is a eukaryotic-specific modification central to many cellular processes, such as cell cycle progression, transcriptional regulation, and hormone signaling. Polyubiquitination is a signal for the 26S proteasome to destroy earmarked proteins, but depending on the polyubiquitin chain topology, it can also result in new protein properties. Both ubiquitin-orchestrated protein degradation and modification have also been shown to be essential for the host's immune response to pathogens. Many animal and plant pathogenic bacteria utilize type III and/or type IV secretion systems to inject effector proteins into host cells, where they subvert host signaling cascades as part of their infection strategy. Recent progress in the determination of effector function has taught us that playing with the host's ubiquitination system seems a general tactic among bacteria. Here, we discuss how bacteria exploit this system to control the timing of their effectors' action by programming them for degradation, to block specific intermediates in mammalian or plant innate immunity, or to target host proteins for degradation by mimicking specific ubiquitin/proteasome system components. In addition to analyzing the effectors that have been described in the literature, we screened publicly available bacterial genomes for mimicry of ubiquitin proteasome system subunits and detected several new putative effectors. Our understanding of the intimate interplay between pathogens and their host's ubiquitin proteasome system is just beginning. This exciting research field will aid in better understanding this interplay, and may also provide new insights into eukaryotic ubiquitination processes.

## Introduction

**Figure 1:**
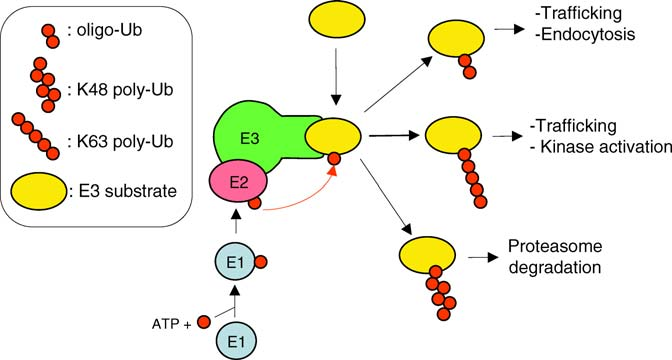
The Eukaryotic UPS Schematic representation of signaling in the UPS, which requires a series of enzymatic steps involving E1, E2, and E3 enzyme complexes that will eventually lead to the addition of Ub moieties to target proteins. The specific recognition of substrates (yellow) by an E3 Ub ligase generally depends on the prior phosphorylation of the substrate (not indicated in the figure). Different types of ubiquitination can lead to different modifications, from proteasome degradation (for the K48-linked poly-Ub chain) to modification in protein properties (K63, oligo Ub [5]). Note that the scheme presented here applies to RING-type E3 ligases; in HECT-type E3s the Ub moiety is transferred from the E2 onto the conserved cystein of the HECT protein, which then transfers this Ub onto the target protein (see text for details).

Ubiquitination is a fundamental post-translational protein modification for all eukaryotic organisms. It controls several critical aspects of cell metabolism, such as cell cycle progression, transcriptional regulation, signal transduction or recognition, and resistance to pathogens [1–3]. Ubiquitination consists of the conjugation of one or several ubiquitin (Ub) moieties onto a target protein (Figure 1). Monoubiquitination can trigger an alteration of the localization and/or the activity of a target protein [4,5]. Polyubiquitination can modulate the properties of the target protein or constitute a signal for its subsequent degradation by the 26S proteasome [6]. As a general rule, the chains comprised of Ub moieties covalently linked together via their lysine residue (K) 48 are earmarked for proteasome-dependent degradation, whereas K63-linked chains are known to activate and modify protein activity and trafficking [4,5]. Cellular proteins can also be modified by a covalent link to Ub-like proteins (e.g., a small ubiquitin-related modifier [SUMO]; NEDD8). These Ub-like modifiers do not form multimeric chains and have been described to modulate protein properties [1,2]. The ubiquitination process involves successive enzymatic activities [4,5,7]: The Ub-activating enzyme (or “E1”) binds to the C-terminus of Ub in an ATP-dependent reaction via a cysteine residue in its active site. The thioester-linked Ub is then transferred to a cysteine residue of the Ub-conjugating enzyme (or “E2”). Different E2 Ub-conjugating enzymes seem to be responsible for the different (K48 and K63) poly-Ub chain topologies [7,8]. Eventually, the Ub ligase enzyme (or “E3”) controls the specificity of substrate ubiquitination by recruiting the target protein. E3 Ub ligases constitute a large protein family present in all eukaryotes and are distributed in two main groups, really interesting new gene (RING)–type, and homologous to E6-AP C-terminus (HECT)–type E3 Ub ligases. RING-type ligases directly and covalently attach the C-terminus of the Ub from an E2 to a lysine residue of the target protein, whereas the HECT-type protein forms a thioester bond with ubiquitin by its active cysteine residue before transferring it to a substrate. RING-type E3 Ub ligases can be either single (U-box type) or multi-subunit enzymes (generally cullin-based) [9]. When a substrate protein is K48-polyubiquitinated (with at least four subunits [10]), it is targeted to the cell proteasome, which unfolds the protein and degrades it into three to 20 residue peptides, which can be further degraded by downstream aminopeptidases [3,11–13]. Although archaeabacteria contain subunits homologous to the 20S proteasome [13], and bacteria contain likely ancestors of the E1 and E2 enzymes [14], the ubiquitination process as such is not found in prokaryotes.

Many Gram-negative pathogenic bacteria of both animals and plants have evolved type III and/or type IV secretion systems (T3/4SSs) as an essential virulence determinant. These secretion systems are large protein complexes spanning the bacterial envelope that are dedicated to the transfer of protein or DNA substrates into target cells to subvert host defense and other signaling cascades for the benefit of the invading pathogen. We refer the reader to some excellent reviews on these topics [15–17]. In the last decade, an enormous body of work has established the role of T3/4SS effector proteins in bacterial virulence. Similarity in structure or function to eukaryotic proteins allows them to interfere with many different cellular processes, including cytoskeleton rearrangement and intracellular trafficking. The biochemical functions they fulfill within the host cell remain undetermined for the plethora of bacterial T3/4SS effectors identified to date. This is a major challenge for understanding the molecular basis of pathogenicity. Recent advances in this field include the discovery of the different T3/4SS effectors of mammalian bacterial pathogens that have the capacity to interfere with the host's Rho GTPase activity, to reorganize the actin cytoskeleton, and to allow or prevent bacterial internalization ([18] and references therein). Another example of the intriguing co-evolution between a pathogen and its host is the type III secretion system (T3SS) effector-mediated suppression of localized programmed cell death, which is triggered in plants when a specific resistance protein recognizes a specific avirulence protein of the pathogen ([19] and references therein).

**Figure 2:**
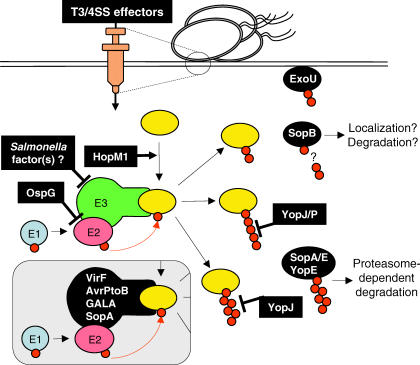
T3/4SS Effectors Interfering with the Host UPS After T3/4SS-mediated translocation into the host cell, several effectors (black-boxes) of diverse bacteria can target different steps of the UPS: some effectors have been shown to inhibit specific steps (e.g., inhibit an E3 Ub ligase, such as OspG, or deubiquitinate subtrates like YopJ and YopP), mimic different UPS E3 Ub ligase subunits (here represented as one black subunit for simplicity as VirF, AvrPtoB, GALA, and SopA), or act as possible adaptor to target substrates to the E3 ligase (HopM1). Eventually, effectors can be a substrate for the UPS, like ExoU, SopB, SopE, and YopE (black ellipses). Table 1 describes in more detail the effectors presented in this figure. “*Salmonella* factor(s)?” refers to unknown factors from non-pathogenic and attenuated *Salmonella* strains (see text).

In this review we focus on the growing number of T3/4SS effectors from both intracellular and extracellular plant and animal bacterial pathogens that specifically exploit their host ubiquitin proteasome system (UPS) (Table 1 and Figure 2). We want to illustrate the different mechanisms that these diverse bacteria have adopted to interfere with this key signaling component of the eukaryotic cell for the benefit of their specific infection strategy. First, we discuss the effectors that utilize the host UPS to ensure their own degradation or modification by ubiquitination, providing a means to regulate their concentration, and for the timing of their action. Second, we present effectors that share the ability to interfere with the ubiquitination level of key cell proteins of mammalian innate immune signaling cascades and, as a result, block the immune response. Third, we discuss two effectors of a plant pathogen that suppress plant innate immune responses, possibly by earmarking host proteins for degradation by the host UPS. Finally, we illustrate the subtle interplay between a pathogen and its host by mimicry of E3 ligase subunits, thereby manipulating the host UPS to the advantage of the pathogen.

**Table 1:**
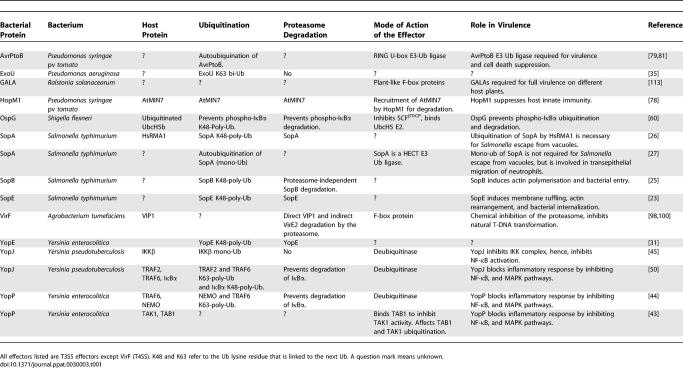
Bacterial T3/4SS Effectors Interfering with Their Host's UPS

## Bacterial Effectors Modulated or Degraded via Ubiquitination by the Host UPS

Recent reports suggest that exploitation of the host UPS could be a general mechanism used by bacteria to program the destruction of a T3/4SS effector when its function in the host cell is no longer required. This could be to temporarily activate a specific host protein or process, or to prevent deleterious effects to the host cell, which needs to stay in an optimal condition for bacterial colonization. The first lines of evidence for such a mechanism come from work on Salmonella enterica serovar Typhimurium *(S. typhimurium)* T3SS effectors. This facultative intracellular bacterium is an important enteric pathogen of humans, causing gastrointestinal inflammation. Two T3SSs, encoded by the *Salmonella* pathogenicity islands 1 (SPI-1) and 2 (SPI-2), are essential for its pathogenicity and are used during different stages of infection for entry into intestinal cells and subsequent replication of the intracellular bacteria, respectively [20,21]. Among the range of translocated T3SS effectors encoded by SPI-1 are two proteins that alter the structure and the function of the actin cytoskeleton but exhibit opposing activities: SopE and SptP. SopE acts as a GTP–GDP (guanosine 5′-triphosphate–guanosine 5′-diphosphate) exchange factor (GEF) that activates the signaling molecules Rac-1 and Cdc42, two proteins of the Rho GTPase family, thus provoking cytoskeleton reorganization, which results in bacterial internalization. In contrast, the SptP effector functions as a GTPase-activating protein that deactivates Rac and Cdc42 [22], allowing the recovery of the actin cytoskeleton's normal appearance a few hours after infection. For successful colonization, the activity of these two T3SS effector proteins has to be temporally regulated within the host cell. The mechanism of this regulation was shown to be due to their differential degradation by the host proteasome. SopE and SptP are delivered in equal amounts during infection, but SopE undergoes polyubiquitination and rapid proteasome-dependent degradation following translocation, whereas SptP is degraded at a much slower rate [23,24].

Two other *Salmonella* SPI-1 T3SS effectors, SopA and SopB, are functionally regulated by host ubiquitination. SopB is a phosphoinositide phosphatase that modulates vesicle trafficking by altering the phosphoinositide metabolism. It was shown to be monoubiquitinated and degraded, although probably not via the proteasome [25]. SopA, a protein required for the elicitation of intestinal inflammation, has been shown to be ubiquitinated within the host cell by the membrane-anchored RING-type E3 Ub ligase HsRMA1, and degraded by the proteasome in an HsMRA1-dependent manner [26]. The authors suggest that HsRMA1-dependent ubiquitination of SopA is involved in the escape of bacteria from *Salmonella*-containing vacuoles into the cytosol of epithelial cells. Recently, the same research group identified SopA as a HECT-type E3 Ub ligase. They identified the catalytic cysteine residue of SopA, and showed the formation of a transient E3-ubiquitin intermediate. These new data are in line with the mono-Ub state of SopA previously detected in the absence of HsRMA1 [26]. Interestingly, this SopA HECT ligase activity is not required for the escape of bacteria from *Salmonella*-containing vacuoles into the cytoplasm, but seems to be involved in *Salmonella*-induced transepithelial migration of polymorphonuclear neutrophils (PMNs) [27]. The recruitment of these inflammatory cells depends on several factors, including interleukin (IL)-8 secretion, and is considered an important factor for the development of *Salmonella*-induced enteritis. The bacterial or host target proteins for the HECT Ub ligase activity of SopA may well be involved in PMN migration, but are unknown at this stage. Together, the data suggest that SopA displays multiple functions during *Salmonella* infection.

Yersinia sp. and Pseudomonas aeruginosa may also modulate the activity of their effectors by a similar strategy. *Yersinia,* the causal agent of plague *(Y. pestis)* and gastrointestinal disorders *(Y. pseudotuberculosis* and *Y. enterocolitica),* is an extracellular pathogen that injects several effectors through its T3SS to provoke disease [28]. Several of these effectors have been shown to interfere with actin cytoskeleton dynamics that are involved in blocking phagocytosis and subsequent bacterial killing. Y. pseudotuberculosis YopE contributes to virulence by inducing depolymerization of actin filaments in the host cells early after contact with Y. pseudotuberculosis via the inhibition of Rho GTPases, which control rearrangements of the actin skeleton. This activity also prevents the formation of pores in the host membranes and subsequent host cell death, and thus enables a prolonged colonization of the host [29,30]. In Y. enterocolitica–infected cells, YopE is polyubiquitinated on lysine K75 and targeted for proteasome degradation [31]. At this time, it is not clear whether the host-mediated YopE destruction is beneficial to the host's defense or to Y. enterocolitica infection. On the one hand, the degradation products of YopE could be a bacterial antigen source for the host to fend off later infections [31,32]. On the other hand, removing YopE could pave the way for YopT (a cysteine protease [33]) and YopO (a kinase [34]), two other T3SS effectors also targeting actin rearrangements in the host cell. Intriguingly, YopE and S. typhimurium SptP both have GTPase-activating protein activity that indirectly inhibits the pathogen-induced actin polymerization, but it is interesting to note that YopE is actively degraded by the host UPS, whereas SptP has a much longer half-life. These two effector proteins, translocated by different pathogens, seem to have evolved a similar strategy for blocking actin polymerization, yet subtle differences in interaction with the host UPS seem to reflect differences in infection strategy.

ExoU is the major T3SS effector of the opportunistic pathogen Pseudomonas aeruginosa and is directly responsible for the death of the infected host cell. This effector has a phospholipase activity inside the host cell, and researchers have recently shown that it is targeted to the host cell membrane and ubiquitinated [35]. ExoU undergoes ubiquitination of a specific lysine residue (K178) by an as yet unknown mechanism. No more than two Ub moieties are added onto K178, and those are mostly via the K63 residue of the first Ub. This modification is not responsible for ExoU activity, plasma membrane location, or toxicity, and has only a minor impact on ExoU stability [35]. The latter point is not surprising considering the length and the topology of this ubiquitination event [5,35], but at this point a role for ubiquitination of ExoU, most likely the result of its membrane localization, is not clear. Interestingly, Y. enterocolitica YopE also has a specific subcellular targeting to the perinuclear membrane, and this property is determined by the amino acids 54 to 75 [36]. Coincidentally, it is the lysine residue K75 that is subjected to this specific ubiquitination, thus suggesting the possibility of an ubiquitination process associated with membrane localization [31].

## Effectors That Interfere with Important Ubiquitination Steps Involved in Mammalian Innate Immune Signaling

**Figure 3:**
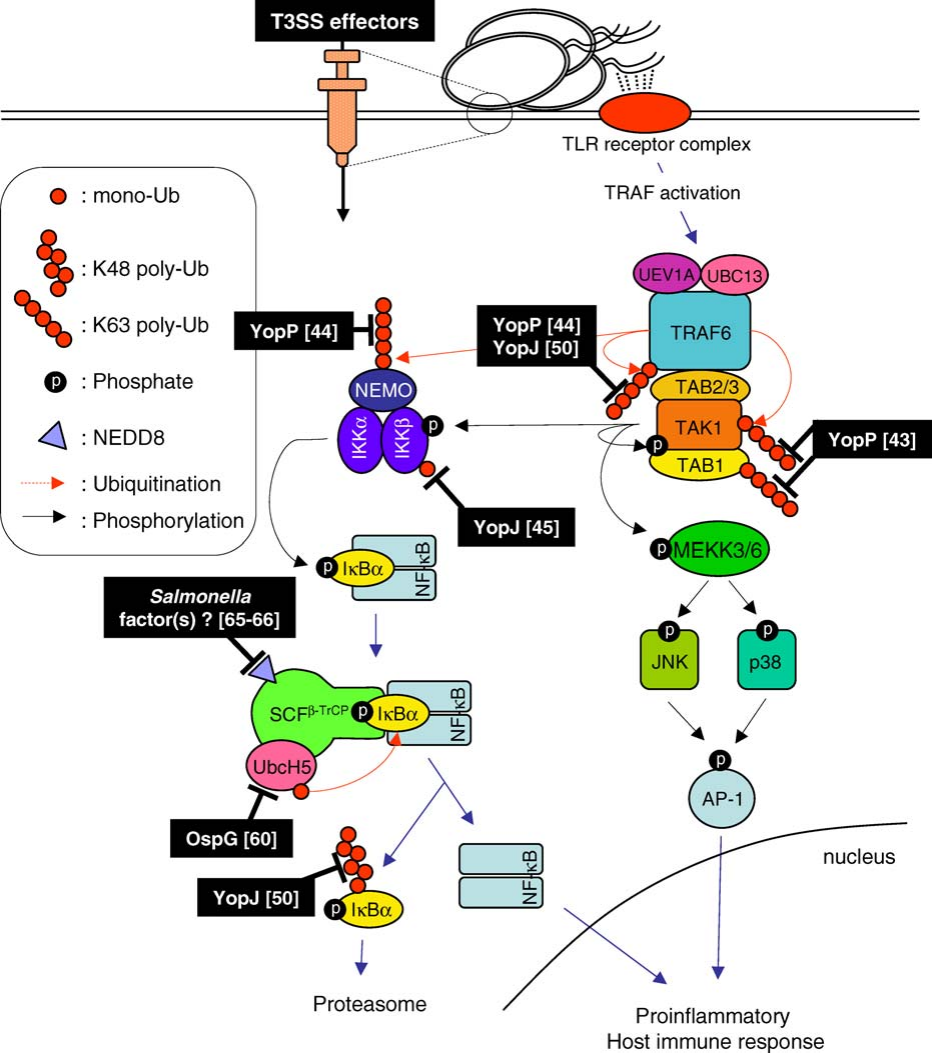
T3SS Effectors Interfering with Ub Signaling in the Mammalian Innate Immune Response Ubiquitination plays an important regulatory role in different steps of the innate immune signaling cascades. This simplified representation shows the different levels at which T3SS effectors (black-boxes) thus far have been shown to interfere with the pro-inflammatory host immune response; this interference can be at the signaling node represented by the TAK1 complex (affecting both NF-κB and the MAPK signaling pathways), or downstream of TAK1, only affecting the NF-κB pathway. Numbers in brackets indicate the specific references. Details of the immune signaling are given in the text.

The innate immune system is the first line of defense in mammals against microbe infection, and it requires several regulatory ubiquitination steps [37]. Several examples have been published of bacterial T3SS effectors that directly interfere with the ubiquitination level of both K48- and K63-linked poly-Ub chains on mediator proteins in the pathogen-induced host defense signaling cascade, thus allowing the bacterium to undermine a proper innate immune response to promote disease. The host's defense mechanism involves receptor-mediated signaling via the mitogen-activated protein kinase (MAPK) and the nuclear factor κB (NF-κB) pathways (we refer the reader to the following reviews [37–40] and Figure 3). Briefly, one of the ways the host can sense pathogens is via perception of microbial-associated molecular patterns (or MAMPs, such as lipoproteins, methylated DNA, lipotechoic acid, flagellin, and lipopolysaccharide) by membrane-anchored Toll-like receptors (TLRs). Pro-inflammatory cytokines, such as tumor necrosis factor α (TNFα) and IL-1, are produced as an alert for the immune system via TLR signaling in response to pathogens, and bind to receptor proteins of the tumor necrosis factor receptor (TNFR) and IL-1 receptor (IL-1R) families. Activated TLRs and IL-1Rs recruit downstream adapter and signaling molecules that are involved in the activation of the crucial signal transducer TNFR-associated factor (TRAF) 6, whereas TRAF2 transduces the signal received by TNFR [37,40,41]. TRAF6 acts as an E3 Ub ligase that recruits the E2 enzyme complex Uev1A/Ubc13 to K63-polyubiquinate itself [42]. This autoubiquitination step allows the recruitment of TGF-β-activated kinase 1 (TAK1)–binding protein (TAB) 2 and TAB3, and subsequently TAK1 and TAB1. After formation of the complex, TRAF6 K63-polyubiquitinates TAK1 [37,38]. Although the precise mechanism is not yet clear, TAB1 also seems to be K63-polyubiquitinated [43]. TAK1 is a highly conserved kinase complex that acts as a central component of the immune response pathway; it can autophosphorylate [42–44] and activate the MAPK (p38, c-Jun NH_2_-terminal kinase) pathway by mitogen-activated protein kinase kinase kinase (MEKK) 3 and MEKK6 phosphorylation, as well as the NF-κB pathway by inhibitor of nuclear factor κB (IκBα) kinase (IKK) β phosphorylation [42]. After this latter modification, IKKβ could be monoubiquitinated by an as yet unknown mechanism [45]. The IKKγ or NFκB essential modifier (NEMO) subunit is also K63-polyubiquitinated by TRAF6, which allows the formation of an active IKK complex from the subunits IKKα, IKKβ, and IKKγ. The transcriptional activator NF-κB is sequestered in the cytoplasm by IκBα and thus is inactive. Phosphorylation of IκBα by the activated IKK complex allows the recognition of IκBα by the E3 Ub ligase SCF^βTrCP^ (Skp1/Cullin1/F-box), which K48-polyubiquitinates IκBα for proteasome degradation. This provokes the release and subsequent nuclear translocation of NF-κB, where it acts as a transcription factor of a large array of target genes, including anti-apoptotic and immune responses genes.

Y. pseudotuberculosis and Y. pestis YopJ, and their homologue YopP in *Y. enterocolitica,* (phrased YopJ/P when we refer to Y. pseudotuberculosis YopJ and Y. enterocolitica YopP proteins at the same time) were shown to interfere with the host inflammatory response via both the MAPK and the anti-apoptotic NF-κB signaling pathways (Figure 3), which prevents the production of pro-inflammatory cytokines such as TNFα and IL-8, and triggers apoptosis. YopJ/P homologues are found in S. typhimurium (protein AvrA) and in some plant pathogenic bacteria [46]. These proteins were assigned as C55 cysteine proteases [47], and by sequence comparison were originally hypothesized to be deSUMOylating enzymes, even though no specific substrate could be identified [48,49]. Recently, different research groups have identified YopJ/P as deubiquitinating enzymes directly involved in the inhibition of the inflammatory response [43–45,50]. Zhou and collaborators showed that Y. pseudotuberculosis YopJ can deubiquitinate the K63-poly-Ub chain of TRAF6 (and TRAF2) inducing the inhibition of both the MAPK and NF-κB pathways. Y. pseudotuberculosis YopJ was also shown to deubiquitinate the K48-poly-Ub chain from IκBα, preventing proteasome-mediated degradation of IκBα and the resulting NF-κB nuclear translocation [50]. In addition, Y. enterocolitica YopP can cleave TRAF6 and NEMO K63-poly-Ub chains, but this activity could only be proven in vitro [44]. Recently, Thiefes and colleagues showed that Y. pestis YopP was not able to deubiquitinate TRAF6, but rather that it cleaved the K63-Ub chains of TAK1 and TAB1 [43]. The versatility of Y. pseudotuberculosis YopJ was further emphasized by the finding that it could remove the single Ub linked to IKKβ [45]. Tampering with Ub in the inflammatory pathway is achieved so efficiently by YopJ of Y. pseudotuberculosis and Yop P of Y. enterocolitica that Y. pestis has evolved a less invasive strategy. Indeed, Y. pestis YopJ is believed to be injected by the T3SS less efficiently than YopP, and, as a consequence, does not result in fast apoptosis of macrophages, but rather weakens the inflammatory response, enabling the pathogen to be transported within the macrophages to target organs [51]. These reports emphasize the crucial role of bacterial effectors in the deubiquitination of key components in the MAPK and NF-κB pathways, on both K48- and K63-poly-Ub chains (Figure 3). Interestingly, the eukaryotic deubiquitinating enzymes cylindromatosis tumor suppressor protein and A20, which are required for cell homeostasis [52–54], can also deubiquitinate the K63-polyubiquitin chains of signaling intermediates of the NF-κB pathway, but are not active on the K48 chains [50,55]. This illustrates that pathogens clearly use mechanisms that are familiar to the host. Even though YopJ/P seem to have an extended deubiquitinating activity (both of K48- and K63-poly-Ub chains), these proteins don't seem to be able to cleave SUMOylated proteins. Indeed, YopJ/P are deubiquitinating enzymes in vitro, with no activity on SUMOylated proteins [50]. The cysteine residue (C172) that was originally defined for the deSUMOylation activity [49] is also required for the deubiquitinating activity [44,50]. We would like to add that recent work shows that Y. pseudotuberculosis YopJ blocks signaling by binding and acetylating residues in the activation loop of MEKK6 (a mitogen-activated protein kinase kinase upstream of c-Jun NH_2_-terminal kinase and p38) [56]. The authors hypothesize that a similar activity could prevent phosphorylation of IKKβ, thus preventing further activation of the NF-κB pathway [56].

Evidently more work is necessary to unravel the mode of action of the YopJ/P family of T3SS effectors. It would be interesting to re-evaluate the function of YopJ/P homologues widely present in plant pathogenic bacteria and currently identified as deSUMOylating enzymes [46,57–59].

The facultative intracellular pathogen *Shigella flexneri,* causal agent of shigellosis in humans, has among its repertoire of T3SS effectors a protein called OspG that is structurally related to a kinase. This protein was shown to be injected into epithelial cells, where it weakens the host innate immune response [60]. OspG negatively regulates the NF-κB inflammatory response by interfering with the proteasome-dependent degradation of IκBα. OspG binds and inhibits ubiquitinated E2 Ub-conjugating enzymes: it interacts with UbcH5, which is necessary for the Ub supply to the E3 Ub ligase SCF^βTrCP^, the specific SCF complex that controls IκBα degradation (Figure 3). OspG displays a kinase activity inducing its autophosphorylation. This activity is necessary for the function of OspG, but doesn't seem to be involved in the phosphorylation of IκBα prior to its K48-polyubiquitination and proteasome-mediated degradation. The mechanism by which OspG inhibits the SCF^βTrCP^-mediated IκBα degradation still has to be elucidated. This T3SS effector is injected into host cells during the early stages of infection [61]. An attractive hypothesis is that the inhibition of the NF-κB inflammatory response in these early stages facilitates cellular colonization by a limited number of luminal bacteria [60]. Considering that OspG can interact with different E2 Ub-conjugating enzymes, this effector might interfere with other UPS targets in the host cells as well.

Non-pathogenic or attenuated *Salmonella* strains (S. typhimurium PhoPc mutant [62,63] and *S. pullorum,* a poultry-specific strain) have also been shown to attenuate the NF-κB-mediated inflammatory response [64]. In doing so, these organisms can thrive in the intestinal microflora. The mechanism by which these strains inhibit the inflammatory response seems to involve the reduction of Cullin1 neddylation. Non-neddylated Cullin1 is still capable of taking part in the SCF^βTrCP^ E3 Ub ligase complex, but could be impaired in the recruitment of the E2 Ub-conjugating enzyme [65,66]. The absence of a functional SCF^βTrCP^ complex would then result in the absence of ubiquitination of phospho-IκBα, hence explaining the observed stabilization of phospho-IκBα. As pointed out by the authors, the bacterial factors responsible for the attenuation of the Cullin1 neddylation haven't been identified yet.

## Effectors Involved in Ubiquitination of Host Proteins to Suppress Plant Innate Immune Responses

Recent work has revealed striking similarities in the response between animals and plants in the recognition of MAMPs, as illustrated by the discovery of a plant receptor reminiscent of TLR5 in humans [67,68], as well as in downstream antimicrobial defense responses that are signaled via MAPK cascades and induction of target gene expression [69,70]. MAMP recognition can initiate basal defense responses, such as strengthening of the cell wall by callose deposition [71,72]. Specific bacterial T3SS effectors are capable of suppressing this basal defense mechanism [73–76]. Another type of resistance, specific for plants, is the hypersensitive response (HR), which causes rapid cell death at the site of infection on resistant plants and thereby prevents bacterial multiplication and spread. This type of resistance is induced by specific recognition of bacterial virulence factors (including T3SS effectors) by cultivar-specific resistance proteins [75]. Also, in the case of HR-mediated resistance, some bacteria have evolved T3SS effectors capable of avoiding this specific type of induced resistance [71,72,75,77–81]. Below, we present two P. syringae T3SS effectors that are capable of suppressing different layers of plant defense, probably by controlling the ubiquitination and degradation of specific proteins by the cell UPS.

P. syringae causes bacterial speck disease on susceptible plants. HopM1 is a P. syringae T3SS effector required for virulence and is known to suppress the plant host cell wall–associated defense [72]. Recently, Nomura and colleagues showed that during bacterial infection of *Arabidopsis* plants, HopM1 mediates the proteasome-dependent elimination of AtMIN7, a plant protein involved in cell wall–associated host defense [78]. Interestingly, HopM1 has no classical E3 Ub ligase features, leading to the hypothesis that HopM1 may act as an adaptor protein mediating the recognition of AtMIN7 by the plant UPS. AtMIN7 is a GEF of the adenosine diphosphate ribosylation factor (ARF) subfamily. ARF-GEFs are important for vesicle trafficking by activation of Ras-like small GTPases [82]. Several lines of evidence show that vesicle trafficking plays an important role in plant immunity [72,83–85]. When challenged with a ΔCEL P. syringae mutant strain (lacking the *hopM1* gene), *AtMIN7* knock-out plants accumulate less callose deposits and are more susceptible to infection than wild-type plants. Altogether, the data suggest that the role of HopM1 in virulence is to inhibit vesicle trafficking associated with cell wall–associated host defense by targeting AtMIN7 for degradation by the host UPS [78].

The *P. syringae* pv *tomato* (strain DC3000) T3SS effectors AvrPto and AvrPtoB both elicit an HR response in tomato plants expressing the *Pto* resistance gene. [86,87]. This rapidly induced localized programmed cell death at the site of infection enables plants to resist colonization by the pathogen. Interestingly, AvrPtoB is also capable of suppressing programmed cell death induced by the AvrPto/Pto recognition in *Nicotiana benthamiana,* and HR elicited by other bacterial T3SS effectors, fungi-specific HR-inducing protein, and even the pre-apoptotic mouse protein Bax [88,89]. AvrPtoB is a modular protein with an N-terminal part that induces HR-related cell death and a C-terminal portion that controls cell death suppression [90]. The C-terminal domain was recently shown to possess the structural features of a RING U-box type E3 Ub ligase [81]. This domain, as well as full-length AvrPtoB, indeed functions as an active E3 Ub ligase capable of autoubiquitination [79,81] and possibly of ubiquitination of plant substrates [79]. The E3 Ub ligase activity is functionally important since mutations impairing the recruitment of the E2 Ub-conjugating enzyme or the autoubiquitination prevent both cell death suppression activity and full virulence of strain DC3000 [79,81]. Even though no target has yet been identified, a possible explanation for the mode of action of AvrPtoB is the specific recognition, ubiquitination, and proteasome-dependent degradation of plant cell death positive regulators. It should be noted here that, in a recent report, AvrPto and AvrPtoB have been identified as potent and early suppressors of MAMP-induced MAPK-dependent innate immunity pathway in *Arabidopsis,* but this function of AvrPtoB is not affected by a mutation that disrupts the E3 Ub ligase activity [74].

## Effectors Mimicking Host E3 Ub Ligases

Mimicking eukaryotic proteins appears to be a strategy commonly used by pathogenic bacteria to promote virulence [91,92]. This can be achieved by convergent evolution, which “produces” a new effector protein with structural characteristics enabling the functional mimicry of a host protein (e.g., AvrPtoB). But this can also be achieved by a more “opportunistic” scenario, in which the bacterial pathogen or one of its ancestors has acquired genetic material by lateral transfer and then maintains and adapts functional domains according to their selective advantage in virulence. The two examples discussed below illustrate the latter scenario and highlight a subtle mechanism bacteria have evolved to directly interfere with plant functions via the UPS.

Agrobacterium tumefaciens causes crown gall disease on a broad range of plants. The bacterium uses a type IV secretion system (T4SS) not only to translocate effectors into eukaryotic cells, but also to mediate the transfer of a single-stranded DNA molecule (transferred [T]-DNA), resulting in genetic colonization of the host [93–95]. VirF is a T4SS effector that determines host range and is necessary for full virulence on certain host plants [96,97]. VirF was the first prokaryotic protein shown to contain a conserved F-box domain [98]. F-box proteins (FBPs) are key components of the SCF type E3 Ub ligase complex, because they recruit the target protein for destruction by the 26S proteasome. Through the F-box domain, FBPs interact with the SKP1 component of the E3 Ub ligase complex [99]. The *Arabidopsis* homologues of the yeast SKP1 protein, ASK1 and ASK2, were isolated as interactors of VirF [98]. The F-box of VirF was shown to be essential not only for this interaction in vitro, but also for virulence. To determine the precise role of VirF in the infection process, recent studies are aimed at identifying the target proteins destined for ubiquitination and possibly degradation by the proteasome. Recently, it was shown that VirF interacts with the plant protein VirE2-interacting protein 1 (VIP1), which leads to degradation of VIP1 and, indirectly, of the effector protein VirE2 [100]. VirE2 is a single-stranded DNA-binding protein that is transported independently from the T-DNA by the T4SS into the host cell [101,102], where it cooperatively binds the T-DNA to facilitate nuclear uptake [103,104] and protect it from degradation [105]. VirE2 contains functional nuclear localization signals [104,106], but these signals overlap with the DNA-binding domain [106,107], which make it difficult to show an in vivo function of the nuclear localization signal region in nuclear uptake of the T-complex. The *Arabidopsis* protein VIP1 was identified as an interactor of VirE2 [108]. This protein was shown to interact with karyopherin-α, a member of the importin family involved in nuclear import of proteins via recognition of their nuclear localization signals. Citovsky and colleagues suggested a role for VIP1 in A. tumefaciens infection as a molecular adaptor between VirE2 and karyopherin-α that results in nuclear uptake of the T-complex [109]. Recently, Tzfira and colleagues proposed that VirF, which binds to VIP1 but not VirE2, is involved in the nuclear proteasome-dependent degradation of VIP1 and indirectly in that of VirE2, and may thus play a role in uncoating the T-complex from VirE2 molecules prior to integration of the T-DNA in the host genome [100]. A host-dependent role in virulence for VirF by indirectly targeting another effector protein for degradation is intriguing; yet, it remains to be determined whether VirF is able to destabilize VIP1 and VirE2 when complexed with T-DNA and, more importantly, during infection. In preliminary experiments, using the C-terminal part of VirF (lacking the F-box domain) as bait in a yeast two hybrid screen, several *Arabidopsis* proteins have been identified as putative targets of VirF (E. Jurado-Jácome, P. Hooykaas, and A. Vergunst, unpublished data). Among these are proteins that have been shown to be involved in host defense–related processes. Although the interaction of some of these proteins has been confirmed in vitro, the relevance of these interactions during infection and their VirF-mediated degradation remains to be confirmed. As described above, a function in disarming host proteins involved in defense against bacterial attack and suppression of the immune response seems to be a general mode of action for effectors of both mammalian and plant pathogens.

The plant pathogen Ralstonia solanacearum uses a T3SS to promote “bacterial wilt” on a variety of plant hosts [110–112]. Among the large repertoire of T3SS effectors identified in this bacterium [110–112] is a family of proteins that is likely to function as eukaryotic FBPs [113]. Indeed, each of the seven members of this effector family harbors both an N-terminal F-box motif for interaction with other subunits of the E3 Ub ligase complex, and a long leucine rich repeat (LRR) domain. A characteristic feature of the LRR is the presence in each of the 24 amino acid–long repeats of conserved residues forming the motif GAxALA, hence the name “GALA” proteins. The structure of these T3SS effector proteins is highly similar to the LRR subclass of plant FBPs [114]. We further showed that GALAs are capable of interacting with several of the 19 *Arabidopsis* SKP1-like proteins (ASKs). Like A. tumefaciens VirF, GALAs interact with ASK1 and ASK2, but also interact with other ASKs, in a manner that is reminiscent of plant FBPs [114,115]. Pathogenicity tests revealed that none of the individual GALA effectors is indispensable for virulence of R. solanacearum on *Arabidopsis* or tomato [112]. The finding that a strain deleted of all seven *GALA* genes is significantly less virulent on tomato and *Arabidopsis* [113] suggests that two or more non-functionally overlapping GALAs are required. Interestingly, when tested on *Medicago truncatula,* another host plant, a single mutant for the *GALA7* gene appears dramatically affected in its virulence. The virulence capacity of this single mutant is restored by complementation with a full length *GALA7* construct, but not by a *GALA7* gene construct deleted of its F-box domain. These results support a model, similar to VirF, in which specific GALAs (GALA7 on Medicago truncatula) and combinations of GALAs could form bacterium/plant composite SCF-type E3 Ub ligases in specific host cells, possibly to ubiquitinate and subsequently degrade mediator(s) of plant defenses.

## Only the Tip of the Iceberg

The bacterial plant pathogens A. tumefaciens and recently R. solanacearum were the first prokaryotes shown to harbor proteins with an F-box that is essential for virulence [98,113]. These FBPs are substrates of T4SS and T3SS, respectively [98,113]. The F-box–containing protein Msi061 of the plant symbiont Mesorhizobium loti was also demonstrated to be transported into plant cells in a heterologous translocation assay by the A. tumefaciens T4SS [116]. The recent completion of the genome sequences of the Legionella pneumophila strains Paris and Lens enabled the annotators to identify three genes that likely encode FBPs, and one gene that encodes a protein with two U-box domains [117], making these proteins attractive candidates for participating in E3 Ub ligases within eukaryotic host cells. The complete genome sequence of many bacteria, including human and plant pathogens, and other bacteria that have close associations with eukaryotes during their life cycle, are now available. Our curiosity about the extent of bacterial effector candidates exploiting the host UPS made us mine the most recent protein database release using the protein domain signature search tool available at InterPro (http://www.ebi.ac.uk/interpro, data release 13.0). We searched for the eukaryotic-specific putative E3 Ub ligase U-box (IPR003613) and F-box (IPR001810) motifs. In addition to the L. pneumophila U-box protein Lpp2887 that was recently annotated [117], we identified one gene encoding a putative U-box domain (locus pc1652) in *Candidatus Protochlamydia amoebophila* UWE25, an obligate endosymbiont of free-living amoebae [118]. In contrast, the search for F-box domains encoded by bacterial genomes was more productive and yielded several new candidates (Table 2). Although InterPro identified the A. tumefaciens VirF protein [98], it only found three out of the seven R. solanacearum GALA proteins [113], and did not detect the Mesorhizobium loti msi061 protein [116], indicating that our screen was not saturating. In *L. pneumophila,* however, our analysis found not only the three already annotated FBPs [117,119], but also three additional FBP candidates (Table 2). *Coxiella burnetii,* the agent of Q-fever [120] closely related to *L. pneumophila,* also contains three genes encoding putative FBPs (Table 2). *Candidatus Protochlamydia amoebophila* UWE25 [118] has numerous (11) proteins containing putative F-boxes. This bacterium is a recently characterized relative of pathogenic *Chlamydia,* but has a much larger genome size, indicating that massive reorganization and genome reduction took place in Chlamydia sp. after divergence in pathogenic and symbiontic *Chlamydia* [118]. The absence of any detectable FBP in pathogenic Chlamydia sp. might be the result of this genome reduction, and the large number of FBPs in UWE25 possibly originate from ancient lateral transfer [118]. This suggests a greater importance of these putative FBPs in symbiosis than in the control of the virulence of its pathogenic relative.

**Table 2:**
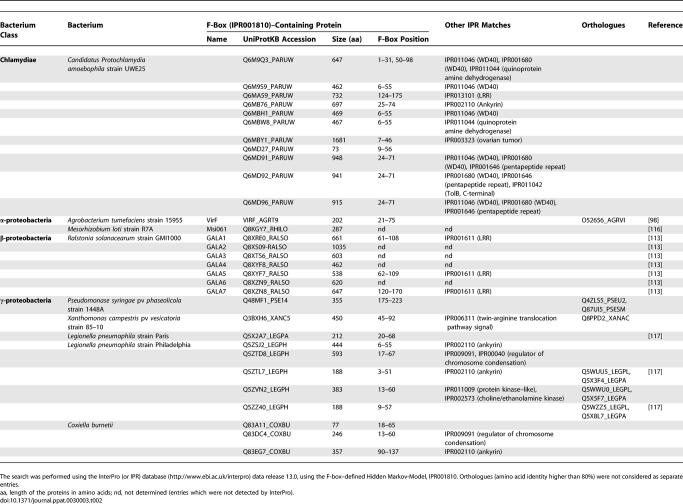
Bacterial F-Box–Containing Proteins

Our screen also revealed conserved F-box–encoding genes among several sequenced plant pathogens, including Xanthomonas sp., and several P. syringae pathovars. In summary, our in silico analysis indicates a wide range of bacteria, from the class of Chlamydiae to α-, β- and γ-proteobacteria, predicted to contain putative FBPs. It will be interesting to find out whether the bacteria with putative FBPs can indeed inject these proteins as substrates of the T3/4SS into host cells, where they could interfere with UPS-controlled mechanisms to benefit in the survival of the pathogen.

Our purpose in this review was to illustrate the different mechanisms used by bacteria to explore the host's UPS by their T3SS or T4SS effectors. To put the exploitation of the UPS by bacterial effectors of T3/4SS into perspective with other mechanisms used by pathogens to interfere with their host's UPS, we would like to just touch on several rapidly expanding groups. Bacterial toxins, transported into host cells by mechanisms other than T3/4SS, have been shown to interfere with the host UPS. Inside the host cell, the subcellular localization of Listeria monocytogenes listeriolysin O and phospholipase C are partially controlled by the host UPS machinery [121,122].The Escherichia coli cytotoxic necrotizing factor-1 toxin induces the permanent activation of host Rho proteins by locking them in a GTP-bound state. These Rho proteins are then rapidly ubiquitinated and degraded by the host cell [123–125]. The overall effect is an increase in Rho activity followed by Rho depletion, resulting in an efficient bacterial internalization and a weaker host inflammatory response [126]. *Rickettsia conorii,* an obligate intracellular pathogen, also seems to require its host UPS for contact-mediated internalisation [127]. Viruses are well known for their ability to subvert their host UPS either by regulating, or by mimicking, host UPS subunits [128–130]. Recent studies suggest that eukaryotic pathogens can also deliver proteins that interfere with the UPS in host cells. Indeed, thanks to a feeding stylet, plant parasitic nematodes can deliver gland-secreted ubiquitin extension proteins potentially interfering with the Ub pathway in plant cells [131,132].

## Conclusion

A number of animal and plant pathogenic bacteria have evolved type III and type IV effectors that, once translocated into the host cell, have the capacity to interfere directly with ubiquitin signaling, a mechanism fundamental to the eukaryotic host cell. These bacteria have developed different strategies to exploit the host cell ubiquitin/proteasome system to their advantage to (i) control the timing of action of their virulence effectors, as exemplified by the Salmonella sp. effectors SopA and SptP, (ii) target specific signaling intermediates involved in mammal or plant innate immunity, as performed by Yersinia sp. YopJ/P, or (iii) mimic specific host-like UPS components, illustrated by the bacterial FBPs.

The variety of examples presented in this review illustrates the effectiveness of pathogens in interfering with host Ub signaling pathways. It also illustrates that each bacterium has developed a different mode of interference with the UPS by its effectors, depending on its infection strategy. Some bacteria suppress their host's immune response (inhibition of innate immune signaling, inhibition of vesicle trafficking), wheras others control the host cell actin cytoskeleton for bacterial internalization. This exciting area of research is advancing at high speed; future research will certainly result in the discovery of more effectors that interfere with the UPS and the identification of specific host targets and the host functions affected. Such discoveries will supplement research on other (eukaryotic and prokaryotic) pathogens and on mechanisms other than T3/4SS that bacteria have evolved to interfere with the host's UPS. It will undoubtedly result in a better understanding of this intimate host–pathogen interaction, as well as provide new insights in eukaryotic ubiquitination processes. In addition, it may also form the basis for the development of a specific new class of antimicrobials. 

## Abbreviations

ARF: adenosine diphosphate ribosylation factor
ASK: *Arabidopsis* SKP1-like protein
FBP: F-box protein
GEF: GDP-GTP exchange factor
GTP: guanosine 5′-triphosphate
HECT: homologous to E6-AP C-terminus
HR: hypersensitive response
IκBα: inhibitor of NF-κB
IKK: inhibitor of NF-κB kinase
IL: interleukin
K: lysine residue
LRR: leucine rich repeat
MAMP: microbial-associated molecular pattern
MAPK: mitogen-activated protein kinase
MEKK: mitogen-activated protein kinase kinase kinase
NEMO: NF-κB essential modifier, or IKKγ subunit
NF-κB: nuclear factor κB
RING: really interesting new gene
SCF: Skp1/Cullin1/F-box, or E3 Ub ligase
SPI: *Salmonella* pathogenicity island
**S. typhimurium,**: *Salmonella enterica* serovar Typhimurium
SUMO: small ubiquitin modifier
T3SS: type III secretion system
T3/4SS: type III and/or type IV secretion system
T4SS: type IV secretion system
TAB: TAK1-binding protein
TAK1: TGF-β-activated kinase 1
T-DNA: transferred DNA
TLR: toll-like receptor
TNFR: tumor necrosis factor receptor
TNFα: tumor necrosis factor α
TRAF: tumor necrosis factor receptor–associated factor
Ub: ubiquitin
UPS: ubiquitin proteasome system
VIP1: VirE2-interacting protein 1

## References

[ppat-0030003-b001] Kerscher O, Felberbaum R, Hochstrasser M (2006). Modification of proteins by ubiquitin and ubiquitin-like proteins. Annu Rev Cell and Dev Biol.

[ppat-0030003-b002] Welchman RL, Gordon C, Mayer RJ (2005). Ubiquitin and ubiquitin-like proteins as multifunctional signals. Nat Rev Mol Cell Biol.

[ppat-0030003-b003] Smalle J, Vierstra RD (2004). The ubiquitin 26S proteasome proteolytic pathway. Annu Rev Plant Biol.

[ppat-0030003-b004] Kirkpatrick DS, Denison C, Gygi SP (2005). Weighing in on ubiquitin: The expanding role of mass-spectrometry-based proteomics. Nat Cell Biol.

[ppat-0030003-b005] Pickart CM, Fushman D (2004). Polyubiquitin chains: Polymeric protein signals. Curr Opin Chem Biol.

[ppat-0030003-b006] Pickart CM, Cohen RE (2004). Proteasomes and their kin: Proteases in the machine age. Nat Rev Mol Cell Biol.

[ppat-0030003-b007] Fang S, Weissman AM (2004). A field guide to ubiquitylation. Cell Mol Life Sci.

[ppat-0030003-b008] Zhang M, Windheim M, Roe SM, Peggie M, Cohen P (2005). Chaperoned ubiquitylation–Crystal structures of the CHIP U box E3 ubiquitin ligase and a CHIP-Ubc13-Uev1a complex. Mol Cell.

[ppat-0030003-b009] Ardley HC, Robinson PA (2005). E3 ubiquitin ligases. Essays Biochem.

[ppat-0030003-b010] Thrower JS, Hoffman L, Rechsteiner M, Pickart CM (2000). Recognition of the polyubiquitin proteolytic signal. EMBO J.

[ppat-0030003-b011] Pines J, Lindon C (2005). Proteolysis: Anytime, any place, anywhere?. Nat Cell Biol.

[ppat-0030003-b012] von Mikecz A (2006). The nuclear ubiquitin-proteasome system. J Cell Sci.

[ppat-0030003-b013] Bochtler M, Ditzel L, Groll M, Hartmann C, Huber R (1999). The proteasome. Annu Rev Biophys Biomol Struct.

[ppat-0030003-b014] Iyer LM, Burroughs AM, Aravind L (2006). The prokaryotic antecedents of the ubiquitin-signaling system and the early evolution of ubiquitin-like beta-grasp domains. Genome Biol.

[ppat-0030003-b015] Cornelis GR (2006). The type III secretion injectisome. Nat Rev Microbiol.

[ppat-0030003-b016] Christie PJ, Cascales E (2005). Structural and dynamic properties of bacterial type IV secretion systems (review). Mol Membr Biol.

[ppat-0030003-b017] Buttner D, Bonas U (2006). Who comes first? How plant pathogenic bacteria orchestrate type III secretion. Curr Opin Microbiol.

[ppat-0030003-b018] Boquet P, Lemichez E (2003). Bacterial virulence factors targeting Rho GTPases: Parasitism or symbiosis?. Trends Cell Biol.

[ppat-0030003-b019] Grant SR, Fisher EJ, Chang JH, Mole BM, Dangl JL (2006). Subterfuge and manipulation: Type III effector proteins of phytopathogenic bacteria. Annu Rev Microbiol.

[ppat-0030003-b020] Waterman SR, Holden DW (2003). Functions and effectors of the *Salmonella* pathogenicity island 2 type III secretion system. Cell Microbiol.

[ppat-0030003-b021] Patel JC, Galan JE (2005). Manipulation of the host actin cytoskeleton by *Salmonella*--All in the name of entry. Curr Opin Microbiol.

[ppat-0030003-b022] Fu Y, Galan JE (1999). A salmonella protein antagonizes Rac-1 and Cdc42 to mediate host-cell recovery after bacterial invasion. Nature.

[ppat-0030003-b023] Kubori T, Galan JE (2003). Temporal regulation of salmonella virulence effector function by proteasome-dependent protein degradation. Cell.

[ppat-0030003-b024] Patel JC, Rossanese OW, Galan JE (2005). The functional interface between *Salmonella* and its host cell: Opportunities for therapeutic intervention. Trends Pharmacol Sci.

[ppat-0030003-b025] Marcus SL, Knodler LA, Finlay BB (2002). Salmonella enterica serovar *Typhimurium* effector SigD/SopB is membrane-associated and ubiquitinated inside host cells. Cell Microbiol.

[ppat-0030003-b026] Zhang Y, Higashide W, Dai S, Sherman DM, Zhou D (2005). Recognition and ubiquitination of *Salmonella* type III effector SopA by a ubiquitin E3 ligase, HsRMA1. J Biol Chem.

[ppat-0030003-b027] Zhang Y, Higashide W, McCormick BA, Chen J, Zhou D (2006). The inflammation-associated *Salmonella* SopA is a HECT-like E3 ubiquitin ligase. Mol Microbiol.

[ppat-0030003-b028] Cornelis GR (2002). The *Yersinia* Ysc-Yop “type III” weaponry. Nat Rev Mol Cell Biol.

[ppat-0030003-b029] Von Pawel-Rammingen U, Telepnev MV, Schmidt G, Aktories K, Wolf-Watz H (2000). GAP activity of the *Yersinia* YopE cytotoxin specifically targets the Rho pathway: A mechanism for disruption of actin microfilament structure. Mol Microbiol.

[ppat-0030003-b030] Viboud GI, Bliska JB (2001). A bacterial type III secretion system inhibits actin polymerization to prevent pore formation in host cell membranes. EMBO J.

[ppat-0030003-b031] Ruckdeschel K, Pfaffinger G, Trulzsch K, Zenner G, Richter K (2006). The proteasome pathway destabilizes *Yersinia* outer protein E and represses its antihost cell activities. J Immunol.

[ppat-0030003-b032] Veiga E, Cossart P (2005). Ubiquitination of intracellular bacteria: A new bacteria-sensing system?. Trends Cell Biol.

[ppat-0030003-b033] Shao F, Merritt PM, Bao Z, Innes RW, Dixon JE (2002). A *Yersinia* effector and a Pseudomonas avirulence protein define a family of cysteine proteases functioning in bacterial pathogenesis. Cell.

[ppat-0030003-b034] Dukuzumuremyi JM, Rosqvist R, Hallberg B, Akerstrom B, Wolf-Watz H (2000). The *Yersinia* protein kinase A is a host factor inducible RhoA/Rac-binding virulence factor. J Biol Chem.

[ppat-0030003-b035] Stirling FR, Cuzick A, Kelly SM, Oxley D, Evans TJ (2006). Eukaryotic localization, activation and ubiquitinylation of a bacterial type III secreted toxin. Cell Microbiol.

[ppat-0030003-b036] Krall R, Zhang Y, Barbieri JT (2004). Intracellular membrane localization of pseudomonas ExoS and *Yersinia* YopE in mammalian cells. J Biol Chem.

[ppat-0030003-b037] Chen ZJ (2005). Ubiquitin signalling in the NF-kappaB pathway. Nat Cell Biol.

[ppat-0030003-b038] Chen ZJ, Bhoj V, Seth RB (2006). Ubiquitin, TAK1 and IKK: Is there a connection?. Cell Death Differ.

[ppat-0030003-b039] Dong C, Davis RJ, Flavell RA (2002). MAP kinases in the immune response. Annu Rev Immunol.

[ppat-0030003-b040] Sumbayev VV, Yasinska IM (2006). Role of MAP kinase-dependent apoptotic pathway in innate immune responses and viral infection. Scand J Immunol.

[ppat-0030003-b041] Dunne A, O'Neill LA (2003). The interleukin-1 receptor/Toll-like receptor superfamily: Signal transduction during inflammation and host defense. Sci STKE.

[ppat-0030003-b042] Wang C, Deng L, Hong M, Akkaraju GR, Inoue J (2001). TAK1 is a ubiquitin-dependent kinase of MKK and IKK. Nature.

[ppat-0030003-b043] Thiefes A, Wolf A, Doerrie A, G AG, Matsumoto K (2006). The *Yersinia enterocolitica effector* YopP inhibits host cell signalling by inactivating the protein kinase TAK1 in the IL-1 signalling pathway. EMBO Rep.

[ppat-0030003-b044] Haase R, Richter K, Pfaffinger G, Courtois G, Ruckdeschel K (2005). *Yersinia* outer protein P suppresses TGF-(beta}-activated kinase-1 activity to impair innate immune signaling in Yersinia enterocolitica-infected cells. J Immunol.

[ppat-0030003-b045] Carter RS, Pennington KN, Ungurait BJ, Arrate P, Ballard DW (2003). Signal-induced ubiquitination of I kappaB Kinase-beta. J Biol Chem.

[ppat-0030003-b046] Hotson A, Mudgett MB (2004). Cysteine proteases in phytopathogenic bacteria: Identification of plant targets and activation of innate immunity. Curr Opin Plant Biol.

[ppat-0030003-b047] Rawlings ND, Morton FR, Barrett AJ (2006). MEROPS: The peptidase database. Nucleic Acids Res.

[ppat-0030003-b048] Orth K (2002). Function of the *Yersinia* effector YopJ. Curr Opin Microbiol.

[ppat-0030003-b049] Orth K, Xu Z, Mudgett MB, Bao ZQ, Palmer LE (2000). Disruption of signaling by *Yersinia* effector YopJ, a ubiquitin-like protein protease. Science.

[ppat-0030003-b050] Zhou H, Monack DM, Kayagaki N, Wertz I, Yin J (2005). *Yersinia* virulence factor YopJ acts as a deubiquitinase to inhibit NF-(kappa}B activation. J Exp Med.

[ppat-0030003-b051] Zauberman A, Cohen S, Manroud E, Flashner Y, Tidhar A (2006). Interaction of Yersinia pestis with macrophages: Limitations in YopJ-dependent apoptosis. Infect Immun.

[ppat-0030003-b052] Brummelkamp TR, Nijman SM, Dirac AM, Bernards R (2003). Loss of the cylindromatosis tumour suppressor inhibits apoptosis by activating NF-kappaB. Nature.

[ppat-0030003-b053] Kovalenko A, Chable-Bessia C, Cantarella G, Israel A, Wallach D (2003). The tumour suppressor CYLD negatively regulates NF-kappaB signalling by deubiquitination. Nature.

[ppat-0030003-b054] Trompouki E, Hatzivassiliou E, Tsichritzis T, Farmer H, Ashworth A (2003). CYLD is a deubiquitinating enzyme that negatively regulates NF-kappaB activation by TNFR family members. Nature.

[ppat-0030003-b055] Wertz IE, O'Rourke KM, Zhou H, Eby M, Aravind L (2004). De-ubiquitination and ubiquitin ligase domains of A20 downregulate NF-kappaB signalling. Nature.

[ppat-0030003-b056] Mukherjee S, Keitany G, Li Y, Wang Y, Ball LH (2006). *Yersinia* YopJ acetylates and inhibits kinase activation by blocking phosphorylation. Science.

[ppat-0030003-b057] Gurlebeck D, Thieme F, Bonas U (2006). Type III effector proteins from the plant pathogen *Xanthomonas* and their role in the interaction with the host plant. J Plant Physiol.

[ppat-0030003-b058] Hotson A, Chosed R, Shu H, Orth K, Mudgett MB (2003). *Xanthomonas* type III effector XopD targets SUMO-conjugated proteins in planta. Mol Microbiol.

[ppat-0030003-b059] Roden J, Eardley L, Hotson A, Cao Y, Mudgett MB (2004). Characterization of the *Xanthomonas* AvrXv4 effector, a SUMO protease translocated into plant cells. Mol Plant Microbe Interact.

[ppat-0030003-b060] Kim DW, Lenzen G, Page AL, Legrain P, Sansonetti PJ (2005). The Shigella flexneri effector OspG interferes with innate immune responses by targeting ubiquitin-conjugating enzymes. Proc Natl Acad Sci U S A.

[ppat-0030003-b061] Demers B, Sansonetti PJ, Parsot C (1998). Induction of type III secretion in Shigella flexneri is associated with differential control of transcription of genes encoding secreted proteins. EMBO J.

[ppat-0030003-b062] Lawley TD, Chan K, Thompson LJ, Kim CC, Govoni GR (2006). Genome-wide screen for salmonella genes required for long-term systemic infection of the mouse. PLoS Pathog.

[ppat-0030003-b063] Rollenhagen C, Bumann D (2006). Salmonella enterica highly expressed genes are disease specific. Infect Immun.

[ppat-0030003-b064] Neish AS, Gewirtz AT, Zeng H, Young AN, Hobert ME (2000). Prokaryotic regulation of epithelial responses by inhibition of IkappaB-alpha ubiquitination. Science.

[ppat-0030003-b065] Read MA, Brownell JE, Gladysheva TB, Hottelet M, Parent LA (2000). Nedd8 modification of cul-1 activates SCF(beta(TrCP))-dependent ubiquitination of IkappaBalpha. Mol Cell Biol.

[ppat-0030003-b066] Collier-Hyams LS, Sloane V, Batten BC, Neish AS (2005). Cutting edge: Bacterial modulation of epithelial signaling via changes in neddylation of cullin-1. J Immunol.

[ppat-0030003-b067] Gomez-Gomez L, Boller T (2000). FLS2: An LRR receptor-like kinase involved in the perception of the bacterial elicitor flagellin in *Arabidopsis*. Mol Cell.

[ppat-0030003-b068] Zipfel C, Kunze G, Chinchilla D, Caniard A, Jones JD (2006). Perception of the bacterial PAMP EF-Tu by the receptor EFR restricts agrobacterium-mediated transformation. Cell.

[ppat-0030003-b069] Asai T, Tena G, Plotnikova J, Willmann MR, Chiu WL (2002). MAP kinase signalling cascade in *Arabidopsis* innate immunity. Nature.

[ppat-0030003-b070] Gomez-Gomez L, Boller T (2002). Flagellin perception: A paradigm for innate immunity. Trends Plant Sci.

[ppat-0030003-b071] Espinosa A, Alfano JR (2004). Disabling surveillance: Bacterial type III secretion system effectors that suppress innate immunity. Cell Microbiol.

[ppat-0030003-b072] DebRoy S, Thilmony R, Kwack YB, Nomura K, He SY (2004). A family of conserved bacterial effectors inhibits salicylic acid-mediated basal immunity and promotes disease necrosis in plants. Proc Natl Acad Sci U S A.

[ppat-0030003-b073] Nimchuk Z, Eulgem T, Holt BF, Dangl JL (2003). Recognition and response in the plant immune system. Annu Rev Genet.

[ppat-0030003-b074] He P, Shan L, Lin NC, Martin GB, Kemmerling B (2006). Specific bacterial suppressors of MAMP signaling upstream of MAPKKK in *Arabidopsis* innate immunity. Cell.

[ppat-0030003-b075] Nurnberger T, Brunner F, Kemmerling B, Piater L (2004). Innate immunity in plants and animals: Striking similarities and obvious differences. Immunol Rev.

[ppat-0030003-b076] Ausubel FM (2005). Are innate immune signaling pathways in plants and animals conserved?. Nat Immunol.

[ppat-0030003-b077] Abramovitch RB, Martin GB (2004). Strategies used by bacterial pathogens to suppress plant defenses. Curr Opin Plant Biol.

[ppat-0030003-b078] Nomura K, Debroy S, Lee YH, Pumplin N, Jones J (2006). A bacterial virulence protein suppresses host innate immunity to cause plant disease. Science.

[ppat-0030003-b079] Abramovitch RB, Janjusevic R, Stebbins CE, Martin GB (2006). Type III effector AvrPtoB requires intrinsic E3 ubiquitin ligase activity to suppress plant cell death and immunity. Proc Natl Acad Sci U S A.

[ppat-0030003-b080] Abramovitch RB, Anderson JC, Martin GB (2006). Bacterial elicitation and evasion of plant innate immunity. Nat Rev Mol Cell Biol.

[ppat-0030003-b081] Janjusevic R, Abramovitch RB, Martin GB, Stebbins CE (2006). A bacterial inhibitor of host programmed cell death defenses is an E3 ubiquitin ligase. Science.

[ppat-0030003-b082] Memon AR (2004). The role of ADP-ribosylation factor and SAR1 in vesicular trafficking in plants. Biochim Biophys Acta.

[ppat-0030003-b083] Hauck P, Thilmony R, He SY (2003). A *Pseudomonas* syringae type III effector suppresses cell wall-based extracellular defense in susceptible *Arabidopsis* plants. Proc Natl Acad Sci U S A.

[ppat-0030003-b084] Bestwick CS, Bennett MH, Mansfield JW (1995). Hrp Mutant of Pseudomonas syringae pv phaseolicola induces cell wall alterations but not membrane damage leading to the hypersensitive reaction in lettuce. Plant Physiol.

[ppat-0030003-b085] Collins NC, Thordal-Christensen H, Lipka V, Bau S, Kombrink E (2003). SNARE-protein-mediated disease resistance at the plant cell wall. Nature.

[ppat-0030003-b086] Kim YJ, Lin NC, Martin GB (2002). Two distinct *Pseudomonas* effector proteins interact with the Pto kinase and activate plant immunity. Cell.

[ppat-0030003-b087] Pedley KF, Martin GB (2003). Molecular basis of Pto-mediated resistance to bacterial speck disease in tomato. Annu Rev Phytopathol.

[ppat-0030003-b088] Abramovitch RB, Kim YJ, Chen S, Dickman MB, Martin GB (2003). *Pseudomonas* type III effector AvrPtoB induces plant disease susceptibility by inhibition of host programmed cell death. EMBO J.

[ppat-0030003-b089] Jamir Y, Guo M, Oh HS, Petnicki-Ocwieja T, Chen S (2004). Identification of Pseudomonas syringae type III effectors that can suppress programmed cell death in plants and yeast. Plant J.

[ppat-0030003-b090] Abramovitch RB, Martin GB (2005). AvrPtoB: A bacterial type III effector that both elicits and suppresses programmed cell death associated with plant immunity. FEMS Microbiol Lett.

[ppat-0030003-b091] Desveaux D, Singer AU, Dangl JL (2006). Type III effector proteins: Doppelgangers of bacterial virulence. Curr Opin Plant Biol.

[ppat-0030003-b092] Stebbins CE, Galan JE (2001). Structural mimicry in bacterial virulence. Nature.

[ppat-0030003-b093] Gelvin SB (2000). *Agrobacterium* and plant genes involved in T-DNA transfer and integration. Annu Rev Plant Physiol Plant Mol Biol.

[ppat-0030003-b094] Cascales E, Christie PJ (2003). The versatile bacterial type IV secretion systems. Nat Rev Microbiol.

[ppat-0030003-b095] Vergunst AC, van Lier MC, den Dulk-Ras A, Stuve TA, Ouwehand A (2005). Positive charge is an important feature of the C-terminal transport signal of the VirB/D4-translocated proteins of *Agrobacterium*. Proc Natl Acad Sci U S A.

[ppat-0030003-b096] Hooykaas PJ, Schilperoort RA (1984). The molecular genetics of crown gall tumorigenesis. Adv Genet.

[ppat-0030003-b097] Melchers LS, Maroney MJ, den Dulk-Ras A, Thompson DV, van Vuuren HA (1990). Octopine and nopaline strains of Agrobacterium tumefaciens differ in virulence; molecular characterization of the virF locus. Plant Mol Biol.

[ppat-0030003-b098] Schrammeijer B, Risseeuw E, Pansegrau W, Regensburg-Tuink TJ, Crosby WL (2001). Interaction of the virulence protein VirF of Agrobacterium tumefaciens with plant homologs of the yeast Skp1 protein. Curr Biol.

[ppat-0030003-b099] Deshaies RJ (1999). SCF and Cullin/Ring H2-based ubiquitin ligases. Annu Rev Cell Dev Biol.

[ppat-0030003-b100] Tzfira T, Vaidya M, Citovsky V (2004). Involvement of targeted proteolysis in plant genetic transformation by *Agrobacterium*. Nature.

[ppat-0030003-b101] Cascales E, Christie PJ (2004). *Agrobacterium* VirB10, an ATP energy sensor required for type IV secretion. Proc Natl Acad Sci U S A.

[ppat-0030003-b102] Vergunst AC, Schrammeijer B, den Dulk-Ras A, de Vlaam CM, Regensburg-Tuink TJ (2000). VirB/D4-dependent protein translocation from *Agrobacterium* into plant cells. Science.

[ppat-0030003-b103] Abu-Arish A, Frenkiel-Krispin D, Fricke T, Tzfira T, Citovsky V (2004). Three-dimensional reconstruction of *Agrobacterium* VirE2 protein with single-stranded DNA. J Biol Chem.

[ppat-0030003-b104] Ziemienowicz A, Merkle T, Schoumacher F, Hohn B, Rossi L (2001). Import of *Agrobacterium* T-DNA into plant nuclei: Two distinct functions of VirD2 and VirE2 proteins. Plant Cell.

[ppat-0030003-b105] Rossi L, Hohn B, Tinland B (1996). Integration of complete transferred DNA units is dependent on the activity of virulence E2 protein of Agrobacterium tumefaciens. Proc Natl Acad Sci U S A.

[ppat-0030003-b106] Citovsky V, Zupan J, Warnick D, Zambryski P (1992). Nuclear localization of *Agrobacterium* VirE2 protein in plant cells. Science.

[ppat-0030003-b107] Dombek P, Ream W (1997). Functional domains of Agrobacterium tumefaciens single-stranded DNA-binding protein VirE2. J Bacteriol.

[ppat-0030003-b108] Tzfira T, Vaidya M, Citovsky V (2001). VIP1, an *Arabidopsis* protein that interacts with *Agrobacterium* VirE2, is involved in VirE2 nuclear import and *Agrobacterium* infectivity. EMBO J.

[ppat-0030003-b109] Citovsky V, Kapelnikov A, Oliel S, Zakai N, Rojas MR (2004). Protein interactions involved in nuclear import of the *Agrobacterium* VirE2 protein in vivo and in vitro. J Biol Chem.

[ppat-0030003-b110] Salanoubat M, Genin S, Artiguenave F, Gouzy J, Mangenot S (2002). Genome sequence of the plant pathogen Ralstonia solanacearum. Nature.

[ppat-0030003-b111] Genin S, Boucher C (2004). Lessons learned from the genome analysis of ralstonia solanacearum. Annu Rev Phytopathol.

[ppat-0030003-b112] Cunnac S, Occhialini A, Barberis P, Boucher C, Genin S (2004). Inventory and functional analysis of the large Hrp regulon in *Ralstonia solanacearum:* Identification of novel effector proteins translocated to plant host cells through the type III secretion system. Mol Microbiol.

[ppat-0030003-b113] Angot A, Peeters N, Lechner E, Vailleau F, Baud C (2006). Ralstonia solanacearum requires F-box-like domain-containing type III effectors to promote disease on several host plants. Proc Natl Acad Sci U S A.

[ppat-0030003-b114] Gagne JM, Downes BP, Shiu SH, Durski AM, Vierstra RD (2002). The F-box subunit of the SCF E3 complex is encoded by a diverse superfamily of genes in *Arabidopsis*. Proc Natl Acad Sci U S A.

[ppat-0030003-b115] Risseeuw EP, Daskalchuk TE, Banks TW, Liu E, Cotelesage J (2003). Protein interaction analysis of SCF ubiquitin E3 ligase subunits from *Arabidopsis*. Plant J.

[ppat-0030003-b116] Hubber A, Vergunst AC, Sullivan JT, Hooykaas PJ, Ronson CW (2004). Symbiotic phenotypes and translocated effector proteins of the Mesorhizobium loti strain R7A VirB/D4 type IV secretion system. Mol Microbiol.

[ppat-0030003-b117] Cazalet C, Rusniok C, Bruggemann H, Zidane N, Magnier A (2004). Evidence in the Legionella pneumophila genome for exploitation of host cell functions and high genome plasticity. Nat Genet.

[ppat-0030003-b118] Horn M, Collingro A, Schmitz-Esser S, Beier CL, Purkhold U (2004). Illuminating the evolutionary history of chlamydiae. Science.

[ppat-0030003-b119] Chien M, Morozova I, Shi S, Sheng H, Chen J (2004). The genomic sequence of the accidental pathogen Legionella pneumophila. Science.

[ppat-0030003-b120] Seshadri R, Paulsen IT, Eisen JA, Read TD, Nelson KE (2003). Complete genome sequence of the Q-fever pathogen Coxiella burnetii. Proc Natl Acad Sci U S A.

[ppat-0030003-b121] Marquis H, Goldfine H, Portnoy DA (1997). Proteolytic pathways of activation and degradation of a bacterial phospholipase C during intracellular infection by Listeria monocytogenes. J Cell Biol.

[ppat-0030003-b122] Schnupf P, Portnoy DA, Decatur AL (2006). Phosphorylation, ubiquitination and degradation of listeriolysin O in mammalian cells: Role of the PEST-like sequence. Cell Microbiol.

[ppat-0030003-b123] Boyer L, Turchi L, Desnues B, Doye A, Ponzio G (2006). CNF1-induced ubiquitylation and proteasome destruction of activated RhoA is impaired in Smurf1-/- cells. Mol Biol Cell.

[ppat-0030003-b124] Doye A, Boyer L, Mettouchi A, Lemichez E (2006). Ubiquitin-mediated proteasomal degradation of Rho proteins by the CNF1 toxin. Methods Enzymol.

[ppat-0030003-b125] Doye A, Mettouchi A, Bossis G, Clement R, Buisson-Touati C (2002). CNF1 exploits the ubiquitin-proteasome machinery to restrict Rho GTPase activation for bacterial host cell invasion. Cell.

[ppat-0030003-b126] Munro P, Flatau G, Doye A, Boyer L, Oregioni O (2004). Activation and proteasomal degradation of rho GTPases by cytotoxic necrotizing factor-1 elicit a controlled inflammatory response. J Biol Chem.

[ppat-0030003-b127] Martinez JJ, Seveau S, Veiga E, Matsuyama S, Cossart P (2005). Ku70, a component of DNA-dependent protein kinase, is a mammalian receptor for Rickettsia conorii. Cell.

[ppat-0030003-b128] Banks L, Pim D, Thomas M (2003). Viruses and the 26S proteasome: Hacking into destruction. Trends Biochem Sci.

[ppat-0030003-b129] Barry M, Fruh K (2006). Viral modulators of cullin RING ubiquitin ligases: Culling the host defense. Sci STKE.

[ppat-0030003-b130] Shackelford J, Pagano JS (2005). Targeting of host-cell ubiquitin pathways by viruses. Essays Biochem.

[ppat-0030003-b131] Gao B, Allen R, Maier T, Davis EL, Baum TJ (2003). The parasitome of the phytonematode Heterodera glycines. Mol Plant Microbe Interact.

[ppat-0030003-b132] Tytgat T, Vanholme B, De Meutter J, Claeys M, Couvreur M (2004). A new class of ubiquitin extension proteins secreted by the dorsal pharyngeal gland in plant parasitic cyst nematodes. Mol Plant Microbe Interact.

